# Clinical Features and Early Recognition of 242 Cases of Autoimmune Encephalitis

**DOI:** 10.3389/fneur.2021.803752

**Published:** 2022-01-13

**Authors:** Mu Yang, Yajun Lian

**Affiliations:** Department of Neurology, The First Affiliated Hospital of Zhengzhou University, Zhengzhou, China

**Keywords:** autoimmune encephalitis, anti-NMDAR encephalitis, anti-GABABR encephalitis, anti-LGI1 encephalitis, rheumatic immunity, autonomic, ankylosing spondylitis

## Abstract

**Objective:** To analyze the clinical features of common autoimmune encephalitis and evaluate the sensitivity of antibodies contributing to focal epilepsy signs and symptoms (ACES) score.

**Methods:** Collecting and analyzing the data of 242 patients with autoimmune encephalitis (AE) diagnosed in the First Affiliated Hospital of Zhengzhou University from August 2015 to December 2020 in this retrospective study. The six items of the ACES score (cognitive symptoms, behavioral changes, autonomic symptoms, speech problems, autoimmune diseases, temporal MRI hyperintensities) were screened in patients with complete clinical data.

**Results:** (1) In total, 242 patients were included, with 147 cases of anti-N-methyl-D-aspartate receptor encephalitis, 47 cases of anti-γ-aminobutyric acid type B (GABA-B) receptor encephalitis, and 48 cases of anti-leucine-rich glioma inactivating protein 1 (LGI1) encephalitis. The most common clinical symptoms are cognitive impairment (77%), behavioral changes (79%), and seizures (71%). In total, 129 cases (54%) combined with autonomic dysfunction, such as gastrointestinal dysmotility, sinus tachycardia, and central hypoventilation. Twelve patients had autoimmune diseases, most of which were of thyroid diseases. (2) One hundred and twenty-seven patients with complete clinical data evaluated ACES score, 126 cases of whom (126/127, 99.2%) were equal to or >2 points, 1 case (1/127, 0.8%) was of <2 points.

**Interpretation:** (1) Cognitive impairment, abnormal behavior, and seizures are the most common manifestations of AE and autonomic symptoms. Thyroid disease is the most autoimmune disease in AE. Clinically, for patients of suspected AE, increasing the knowledge and testing of thyroid function and rheumatism is necessary. (2) ACES score is a simple, effective, and easy-to-operate score, with a certain screening value for most patients suspected of AE.

## Introduction

Since the first case of anti-N-methyl-D-aspartate receptor (NMDAR) encephalitis was reported in 2007 (1), more and more autoimmune antibodies have been discovered, and autoimmune encephalitis (AE) has gradually become well-known. Most patients with rapidly progressing brain symptoms can be identified quickly. Still, some patients with mild manifestations and no obvious encephalitis symptoms or only presented as seizures are likely to miss and delay treatment. To identify AE at an early stage, Bruijn et al. (2) conducted a prospective, multi-center, observational study in which 582 patients with unexplained focal epilepsy, and no obvious symptoms of encephalitis were tested for antibodies. Twenty patients were positive for autoimmune antibodies (GAD65 *n* = 13, LGI1 *n* = 3, CASPR2 *n* = 3, NMDAR *n* = 1), and the clinical symptoms of the 20 patients were analyzed and the antibodies contributing to focal epilepsy signs and symptoms (ACES) score was obtained. The six items include cognitive symptoms, behavioral changes, autonomic symptoms, speech problems, autoimmune diseases, and temporal MRI hyperintensities. Each factor is assigned 1 point. Antibody testing is recommended when the score is ≥2 points for which the sensitivity is 100%, specificity is 84.9%, and they are highly suspected as the autoimmune causes of epileptic seizures. To verify the sensitivity of the ACES score, 242 definite cases with AE were retrospectively collected, and clinical features of which were analyzed. At last, 127 cases with complete data were selected to validate the score.

## Materials and Methods

In this retrospective study, 242 patients with definite AE in the First Affiliated Hospital of Zhengzhou University were enrolled in this study from August 2015 to December 2020, which includes 147 cases of anti-NMDAR encephalitis, 47 cases of anti-GABA_B_R encephalitis, and 48 cases of anti-LGI l encephalitis. All patients with AE were identified via their medical records. During hospitalization, all the manifested symptoms were observed and recorded. We analyzed their clinical data, such as the demographic characteristics (age and sex), prodromal symptoms (headache, fever, cough, etc.), clinical manifestations (disorders of memory, behavior, speech problems, epilepsy, autonomic system, etc.), co-existing autoimmunity, tumors, previous history, previous and current medication, and modified Rankin Score (mRS). The auxiliary examination included routine and biochemical tests of serum and cerebrospinal fluid (CSF), tests of the virus, thyroid function, tests for rheumatic diseases, 3.0 T brain MRI scanner (Siemens, Germany), CT scan of the thorax, ultrasound of the abdomen, pelvic and reproductive regions, routine electroencephalogram (EEG), and/or Video EEG monitoring. The *J Neurol Neurosurg Psychiatry* (2021) was the diagnostic criterion for AE (3).

Several encephalitis-associated antibodies were screened, such as NMDAR, γ-aminobutyric acid type B **(**GABA-B), contactin-associated protein-2 (CASPR2), leucine-rich glioma inactivating protein 1 (LGI1), and α-amino-3-hydroxy-5-methyl-4-isoxazole propionic acid receptor (AMPAR). All patients were positive for the neuronal antibody in the serum and/or CSF. Cell-based assays (CBAs) for clinical laboratory studies were conducted using Euroimmun IIFT kits: Autoimmune Encephalitis Mosaic 1 (FA 1121-1005-1), and/or NMDAR kits (FA112d-1005-51), based on the instructions provided by the manufacturer, which is highly sensitive and specific assay. Tissue-based assays (TBAs, Euroimmun, Germany) are used in confirmative tests in addition to CBA if CSF is not available but the serum is available, or serum but not CSF is positive.

## Results

### Selected Patients

**Figure 1:**
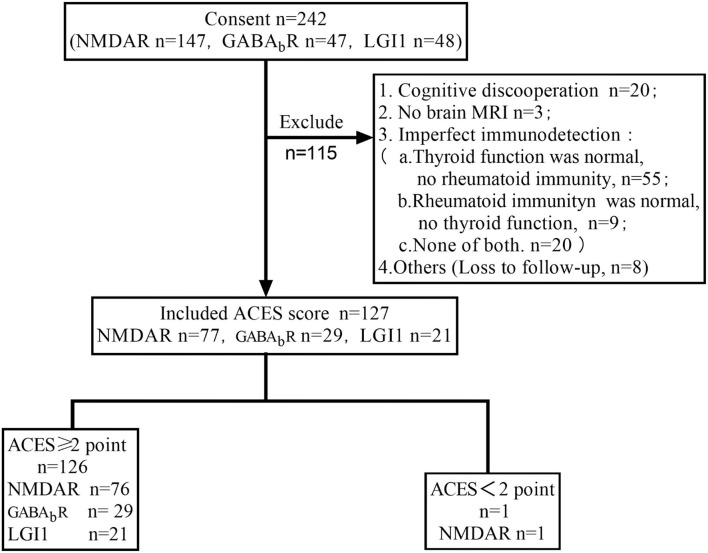
Study flowchart.

In total, 242 patients were screened out, of which 127 patients had complete data of 6 items in ACES score were calculated score (Figure 1).

### Clinical Features of 242 Patients With AE

**Figure 2:**
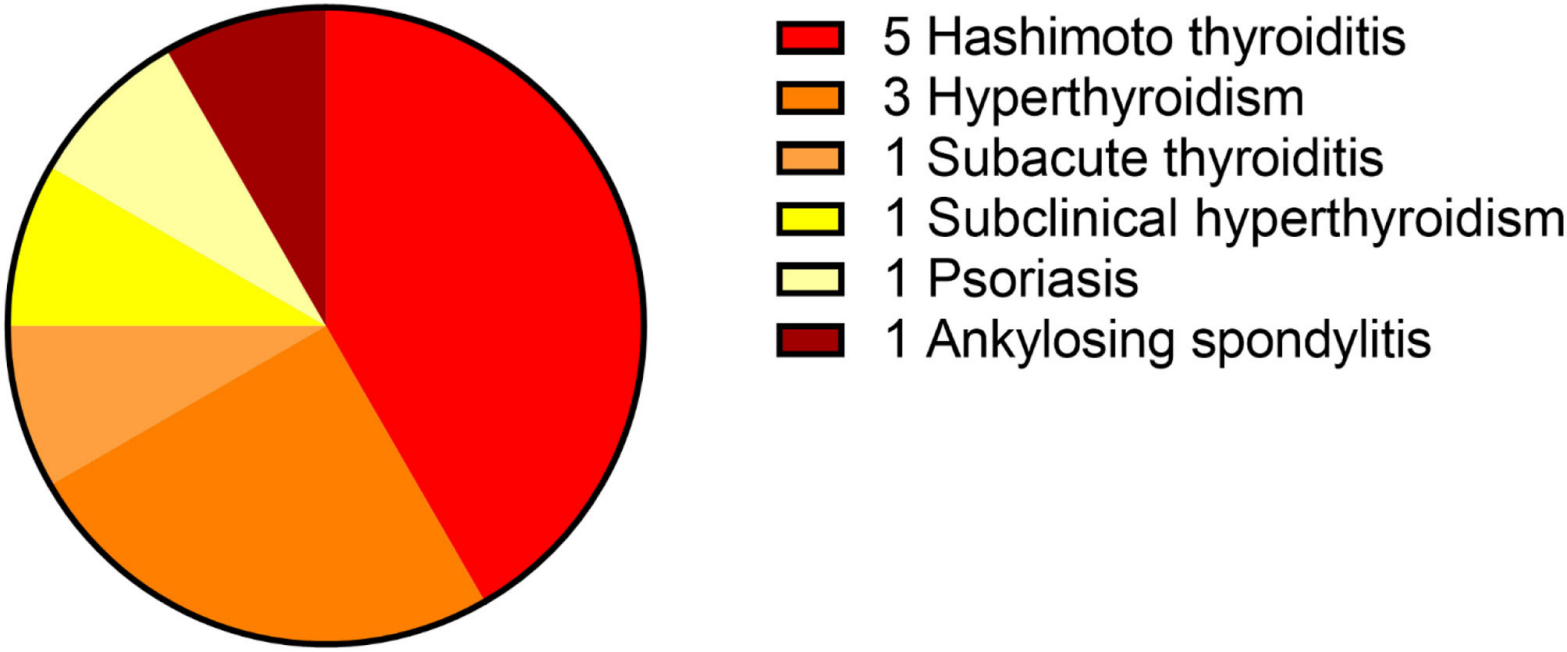
Twelve cases of AE complicated with autoimmune diseases. AE, autoimmune encephalitis.

**Figure 3:**
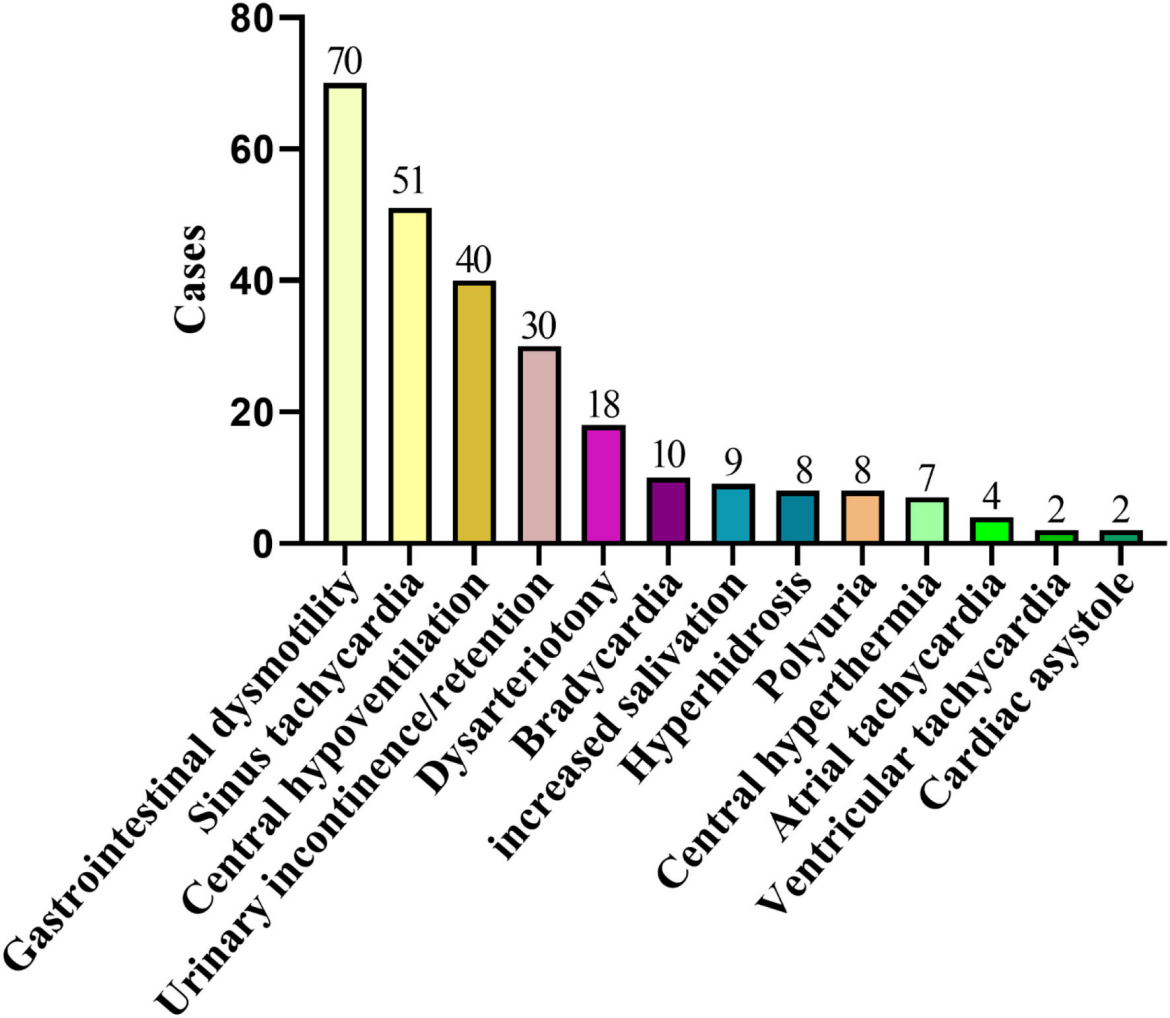
Two hundred and twenty-nine cases of AE complicated with autonomic nerve dysfunction. AE, autoimmune encephalitis.

**Figure 4:**
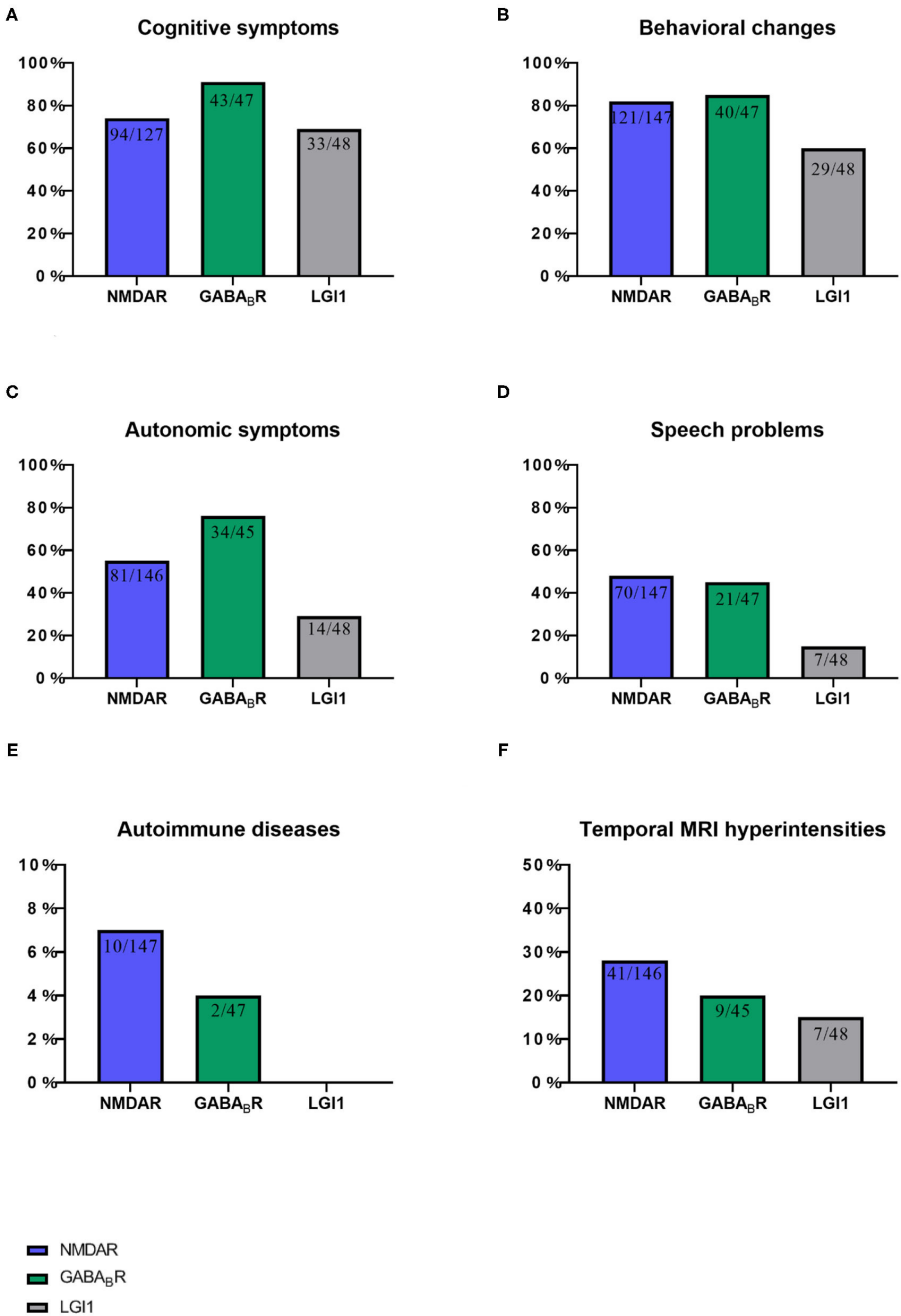
This chart shows the proportion of six items of ACES score in three autoimmune encephalitis. **(A)** Cognitive symptoms, **(B)** Behavioral changes, **(C)** Autonomic symptoms, **(D)** Speech problems, **(E)** Autoimmune diseases, and **(F)** Temporal MRI hyperintensities. NMDAR, N-methyl-D-aspartate receptor; GABABR, aminobutyric acid type B; LGI1,leucine-rich glioma-inactivated 1.

This study included 242 definite patients with AE, 147 patients were anti-NMDAR encephalitis, accounting for 61%. Among them, 100 patients had prodrome, such as fever (*n* = 63), headache (*n* = 63), vomiting (*n* = 25), dizziness (*n* = 25), fatigue or drowsiness (*n* = 25), bloating, diarrhea and poor appetite (*n* = 25), cough (*n* = 5), and sore throat (*n* = 5). Ninety-four patients had cognitive problems, 121 patients had mental and behavior changes, 96 patients had seizures, 81 patients had autonomic symptoms (Figure 2), 70 patients had speech problems, and 41 patients had temporal MRI hyperintensities. Ten patients had other immune diseases (IDs), such as Hashimoto's thyroiditis (*n* = 5), hyperthyroidism (*n* = 5), subclinical hyperthyroidism (*n* = 1), subacute thyroiditis (*n* = 1), and psoriasis (*n* = 1; Figures 3, 4). Fifty-seven cases were virus seropositive and 84 cases were admitted to the ICU. Sixteen cases combined with tumors, such as teratomas (*n* = 8), lung cancer (*n* = 2), renal hamartoma (*n* = 1), sigmoid colon cancer (*n* = 1), meningioma (*n* = 1), gallbladder cancer (*n* = 1), and nasal myofibroblastoma (*n* = 1; Table 1).

**Table 1:** Clinical characteristics of 242 cases of AE.

	**NMDAR**	**GABABR**	**LGI1**
	**(*n* = 147)**	**(*n* = 47)**	**(*n* = 48)**
Median age, range (y)	30 (9 m−83 y)	60 (15–90)	61.5 (14–73)
Sex ratio (F:M)	73:77 (1:1.05)	18:29 (1:1.61)	11:37 (1:3.4)
Prodrome	100/147 (68%)	23/47 (49%)	10/48 (21%)
Cognitive problems	94/127 (74%)	43/47 (91%)	33/48(69%)
Mental behavior change	121/147(82%)	40/47 (85%)	29/48 (60%)
Autonomic symptoms	81/146 (55%)	34/45 (76%)	14/48 (29%)
Seizures	96/147 (65%)	39/47 (83%)	37/48 (77%)
Focal	27/96 (28%)	8/39 (21%)	11/48 (23%)
General tonic clonus	80/96 (83%)	34/39 (87%)	27/48 (56%)
Status epilepticus	41/96 (43%)	8/39 (21%)	2/48 (4%)
Speech problems	70/147 (48%)	21/47 (45%)	7/48 (15%)
Temporal MRI hyperintensities	41/146 (28%)	9/45 (20%)	7/48 (15%)
Other immune diseases	10/147 (7%)	2/47 (4%)	0/48 (0%)
Abnormal TF and rheumatism	10/72 (14%)	4/21 (19%)	2/22 (9%)
Abnormal TF	35/117 (30%)	13/38 (34%)	9/44 (20%)
Abnormal Rheumatism	28/84 (33%)	10/24 (42%)	3/24 (13%)
Virus infection, *n* (%)	57/87 (66%)	20/28 (71%)	6/17 (35%)
Admission to the ICU, *n* (%)	84/146 (58%)	26/47 (55%)	4/48 (8%)
Tumors, *n* (%)	16/147 (11%)	18/47 (40%)	0/48 (0%)

*Positive cases/available cases (percentage)*.

*ICU, intensive care unit; AE, autoimmune encephalitis; ACES, antibodies contributing to focal epilepsy signs and symptoms; TF, thyroid function*.

Among the 47 patients of anti-GABA_B_R encephalitis, 23 patients had prodrome, such as fever (*n* = 9), headache (*n* = 10), vomiting (*n* = 7), dizziness (*n* = 6), bloating, diarrhea and poor appetite (*n* = 5), fatigue or drowsiness (*n* = 4), cough (*n* = 1), and sore throat (*n* = 1). Forty-three patients had cognitive problems, 40 patients had mental and behavior changes, 39 patients had seizures, 34 patients had autonomic symptoms (Figure 2), 21 patients had speech problems, and 9 patients had temporal MRI hyperintensities. Two patients had autoimmune diseases, such as hyperthyroidism (*n* = 1), and ankylosing spondylitis (*n* = 1; Figures 3, 4). Twenty cases were virus seropositive. Nineteen cases combined with tumors, such as lung cancer (*n* = 15; 10 cases were small cell lung cancer), urothelial carcinoma (*n* = 1), and esophageal cancer (*n* = 1).

Among the 48 patients of anti-LGI1 encephalitis, 10 patients had prodrome, such as fever (*n* = 2), headache (*n* = 2), dizziness (*n* = 8), and fatigue or drowsiness (*n* = 2). Thirty-three patients had cognitive problems, 29 patients had mental and behavior changes, 37 patients had seizures, 14 patients had autonomic symptoms (Figures 2, 4), 7 patients had speech problems, and 7 patients had temporal MRI hyperintensities. There were no patients who had an autoimmune disease. Six cases were tested for virus seropositive. Four cases were admitted to the ICU, and there were no patients with tumors.

### ACES Score of 127 Patients With AE

There were 127 patients with a full set of data were selected to take the ACES score test (Table 2).

**Table 2:** ACES scores of 127 cases of AE.

	**NMDAR (*n* = 77)**	**GABABR (*n* = 29)**	**LGI1 (*n* = 21)**
ACES score	77/147 (53%)	29/47 (62%)	21/48 (44%)
4–6	28/77 (36%)	12/29 (41%)	2/21 (10%)
2–3	48/77 (62%)	17/29 (59%)	19/21(90%)
<2	1/77 (3%)	0/30 (0%)	0/22 (0%)

*AE, autoimmune encephalitis; ACES, antibodies contributing to focal epilepsy signs and symptoms*.

As shown in Table 2, 77 patients (77/147, 53%) with anti-NMDAR encephalitis are examined for ACES score. Among them, 28 cases (28/77, 36%) scored ≥4 points, 48 cases (48/77, 62%) scored 2–3 points, and one case scored <2 (1/77, 3%) points. Seventy cases were excluded, among whom 49 cases were due to lack of information on immunological tests. In addition, in those 49 cases, 13 cases were not tested for either thyroid function or rheumatism, seven cases were only normal for rheumatism test, and 28 cases were normal in thyroid function alone. Furthermore, 20 patients were not able to cooperate, as most of them were in a coma state when admitted into the hospital. In addition, one case did not have a brain MRI, and one case was lost to follow-up.

Twenty-nine patients (29/47, 62%) with anti-GABA_B_R encephalitis were examined for ACES scores. Among them, 12 cases (12/29, 41%) scored ≥4 points, 17 cases (17/29, 59%) scored 2–3 points, and all patients scored ≥2 points. Eighteen cases were excluded, among whom 5 cases were not tested for either thyroid function or rheumatism, nine cases were only normal for thyroid function but did not undergo rheumatism tests, 2 cases had no brain MRI, and two cases were lost to follow-up.

Twenty-one patients (21/48, 44%) with anti-LGI1 encephalitis were examined for ACES scores. Among them, 2 cases (2/21, 10%) scored ≥ 4 points, 19 cases (19/21, 90%) scored 2–3 points, and all patients scored ≥2 points. Twenty-seven cases were excluded, among which 18 cases were only tested for thyroid function, 2 cases were only tested for rheumatism, 2 cases were not tested for either thyroid function or rheumatism, and five cases were lost to follow-up.

### One Patient With a Score of ACES <2 Points

The patient was admitted to the hospital with generalized tonic-clonic seizures, fever (37.4–38.4°C), and headache. Eight days before admission, the patient caught a cold and felt uncomfortable. Then the patient had three seizures in a week. The patient had no signs and symptoms of cognitive impairment and abnormal behaviors. The patient was treated with lumbar puncture, routine EEG, brain MRI, and other examinations upon admission. The patient was given antiviral and antiepileptic treatment initially, but the patient still had intermittent headaches. When the tests for CSF autoimmune antibody showed anti-NMDAR antibody was positive, the patient underwent first-line immunotherapy of intravenous methylprednisolone 80 mg daily for 2 weeks. Symptoms of the patient gradually improved and the headache disappeared. Auxiliary examination showed the EEG: abnormal electroencephalogram, diffuse seita-based abnormal slow-wave activity, brain MRI: swelling of the right temporal lobe with abnormal enhancement of pial, inflammation, and chest CT: subpleural inflammation in the right lower lobe, nodular thickening of the right lung pleura. The pressure of CSF was 160 mm H_2_O, the white blood cell count was 52 × 10^6^, and the protein was 362.5 mg/L. The titer of the CSF anti-NMDAR antibody was 1:3.2. Two months after he came back, the CSF antibody became negative. The test result showed that white blood cells were 4 × 10^6^, the protein was 453.1 mg/L. There was no seizure and no discomfort at the follow-up at 4 months.

## Discussion

There has been an increasing knowledge of AE. One study in Minnesota reported an estimated incidence of AE in the United States of America, of 0.8/100,000 person-years (17). In contrast, the incidence of encephalitis in adults described from the western world varies 0.7–12.6/100,000 person-years (18). This difference could be explained by age difference and may be related to the improved awareness of clinicians and the expansion of the detection range (19). This article retrospectively collected and analyzed the clinical characteristics of common clinical AE, scored and verified 130 AE with complete data based on the six items of the ACES score.

Early recognition of AE is essential. A prospective, multi-center, population-based study shows that autoimmunity has become the third most common cause of encephalitis (20). A California study showed that in patients with encephalitis under the age of 30, the frequency of anti-NMDAR encephalitis has exceeded that of viral encephalitis (21). In clinical, anti-NMDAR encephalitis, anti-GABA_B_R encephalitis, and anti-LGI1 encephalitis were most the common AE types, which were consistent with the study by Guy et al. these three types of encephalitis account for 80.95, 7.41, and 4.76%, respectively (22). The main clinical symptoms in this article are cognitive problems (77%), behavioral changes (79%), and epileptic seizures (71%). The results are consistent with the other previous studies (23, 24).

In addition to the widely known symptoms, autonomic symptoms are often diverse and easily overlooked (Table 3). AE usually involves the limbic system (19), where is the center of the autonomic nerve for important visceral activities, such as breathing, heartbeat, and gastrointestinal function (25). Therefore, the clinical features are varied. Bozzetti et al. (11) listed common autonomic symptoms, such as sustained atrial tachycardia or bradycardia, orthostatic hypotension, hyperhidrosis, persistently labile blood pressure, ventricular tachycardia, cardiac asystole, or gastrointestinal dysmotility when verifying the APE2 and RITE2 scores, not caused by drugs, hypovolemia, plasma exchange, or infection, which were scored in the absence of a history of autonomic dysfunction. In addition, the Chinese consensus also mentioned symptoms, such as sinus tachycardia, increased salivation, central hypoventilation, and Central hyperthermia (4). The cases reported by Shin et al. (13) cited constipation and urinary incontinence. In 2017, in the study of Mayo Clinic et al. (14), about 25% of patients with anti-LGI1 and CASPR2 antibodies had autonomic symptoms of which orthostatic hypotension and reduced sweating were the most common symptoms. Besides, gastrointestinal symptoms, such as early satiety, nausea, and constipation, were included. In this study, 127 cases (127/245, 52%) had autonomic symptoms, among which gastrointestinal motility and sinus tachycardia were the most common autonomic symptoms. However, 69% of anti-NMDAR encephalitis studied by Dalmau et al. (12) (55% of NMDAR in this paper) had autonomic nervous function instability, which shows that some autonomic symptoms might have been missed because there is still a certain lack of clinical understanding of it. Therefore, it can be seen that autonomic symptoms are common in AE. In recent years, we have experienced a process of understanding, learning, and improving AE. After knowing the score, more attention will be paid to the autonomic symptoms. We will consider it as a part of developing AE and increasing the suspicion of AE with autonomic symptoms in clinical.

**Table 3:** ACES scores and symptoms of 127 patients with AE.

**ACES score**	**Related symptoms previously reported in the literature**	**This article**
Cognitive problems	Recent memory decline, etc. (4)	170/222 (77%)
Behavioral changes	Anxiety, bizarre, agitation (5), obsessive–compulsive behavior (6), suicidality (7), depression, hallucination (8), catatonia (9), anorexia nervosa (10), babble, apathy (this paper)	190/242 (79%)
Autonomic symptoms	Sustained atrial tachycardia or bradycardia, orthostatic hypotension, hyperhidrosis, persistently labile blood pressure, ventricular tachycardia, cardiac asystole or gastrointestinal dysmotility (11) [Paralytic Ileus (12)], increased salivation, sinus tachycardia, sinus bradycardia, hypotension, Central hyperthermia, Central hypoventilation, hypothermia, etc. (11), constipation, urinary incontinence (13), early satiety, nausea (14)	129/239 (54%)
Speech problems	Difficulty in finding words, speech dysfluency (2), and silent (4)	98/242 (40%)
Other immune diseases	Hashimoto's thyroiditis, systemic lupus erythematosus, anaphylactoid purpura, vitiligo, Sjogren's syndrome, uveitis, myasthenia gravis, chronic urticaria, bullous pemphigoid (15), type I diabetes, Crohn's disease (2), psoriasis (16), ankylosing spondylitis (this paper)	12/242 (5%)
Temporal MRI hyperintensity	–	57/239 (24%)

*AE, autoimmune encephalitis; ACES, antibodies contributing to focal epilepsy signs and symptoms*.

There were also many reports on AE combined with other IDs (Table 3). Among the 20 confirmed AE included by de Bruijn et al. (2), 1 of 7 patients with extracellular antigens (LGI1, CASPR2, NMDA) and 8 of 13 patients with GAD65 had IDs. Of the 517 patients with AE reported by Zhao in 2019, 45 patients had IDs, such as Hashimoto's thyroiditis (*n* = 28), systemic lupus erythematosus (SLE; *n* = 3), anaphylactoid purpura (*n* = 3), vitiligo (*n* = 3), Sjögren's syndrome (*n* = 2), chronic urticaria (*n* = 2), bullous pemphigoid (*n* = 1), uveitis (*n* = 1), myasthenia gravis (*n* = 1), and the coexistence of SLE and anaphylactoid purpura (*n* = 1) (15). In 2019, Qian et al. (16) reported a patient with 30 years of psoriasis that was positive for both anti-CASPR2 and anti-LGI1 antibodies. Haitao et al. (26) reported 3 cases (2 cases of LGI1, 1 case of anti-IgLON5 encephalopathy) of patients with AE with vitiligo. Apart from the overlapped IDs mentioned above, a patient with ankylosing spondylitis, which has not been reported before, was studied in this paper. As is well-known, Interleukin-17 (IL-17) plays an important role in the rheumatic immune system (27). In animal experiments, the absence of IL-17 receptors can avoid collagen-induced rheumatoid arthritis and AE (28), suggesting that AE and IDs may have the same mechanism. Moreover, abnormal thyroid antibodies are common in anti-NMDAR encephalitis and other IDs, such as multiple sclerosis (29, 30). In this study, 83 (83/146, 57%) patients showed immune abnormalities, among them 50 (50/125, 40%) patients had abnormal thyroid function. In addition, consistent with other studies, 10 out of 12 cases (83%) were thyroid diseases. Additionally, immune data from 99 of the 244 (99/244, 40.57%) patients with AE were still incomplete or missing and were excluded when verifying the ACES score. It can be seen that comprehension is needed in clinical understanding and early detection of AE-related IDs. Therefore, thyroid function and rheumatism should be tested routinely in clinical practice to prevent missed diagnoses.

Viral infection may be a potential cause of AE (31). Some studies have shown that potential mechanisms by which infection can lead to breaking of CNS immune tolerance are manifold and include molecular mimicry, change in antigen expression, alternative splicing, post-translational modification, covalent modification, enzymatic processing, protein misfolding, unmasking of cryptic neural epitopes, dysregulation of immune regulators, bystander activation, and “epitope spreading” in the infectious microenvironment (32). A prospective study showed that 27% of Herpes simplex virus encephalitis (HSE) patients are developed into AE (33). In the animal experiments studied by Linnoila et al. (34), more than half of the mice vaccinated with HSV-1 produced anti-NMDAR antibodies. In this article, 84 patients (84/124, 68%) were positive for serovirus, which indicated that viral infection might be a potential cause of AE. If viral encephalitis can be identified and treated early in clinical, it may be prevented from developing into AE. Considering the breaking of the virus to the CNS, antiviral agents may play a role in the prognosis of AE and still need further study.

This study reveals that 127 patients were scored, of which 126 patients (126/127, 99.2%) had ≥ 2 points, 1 case (1/127, 0.8%) had <2 points (NMDA *n* = 1). This patient scored one point had generalized tonic-clonic seizures that did not meet the inclusion conditions of the ACES score, which shows that the results of this article do not conflict with the original text. In this study, 99.2% of patients had a score of ≥2, which is not consistent with all patients (20/20, 100%) in the original text with a score of ≥2, which may be related to the inclusion conditions. The object of the de Bruijn Marienke AAM's study had focal epilepsy of unknown cause without obvious symptoms of encephalitis, and the number of samples is small (*n* = 20). This article expanded the scope of the study (*n* = 127) and included all patients with confirmed AEs, but some patients had no seizures or showed general tonic-clonic seizures. In this study, 99.2% of patients were no <2 points. This indicates that the ACES score is still a simple, reliable, and easy-to-operate screening method for patients with AE.

However, more works need to be done. A prospective study of the reliability of the ACES score is necessary. The study by Hansen et al. (35) showed that there are various manifestations of psychiatric symptoms in AE. Given that the behavior is a broad term, it is important to develop a better scoring system that includes different aspects of psychiatric symptoms.

This study has several limitations. First, it was a retrospective analysis, yet parts of the biochemical data were not available in some cases, such as thyroid function and rheumatism. Second, recall bias might emerge as the result of the long time span and loss of follow-ups during the collection. Third, the range of AE selected was small, but it might not possible to screen all types of AE. Naturally, there are advantages. Firstly, the sample size was large. The AE validated in this article is common in clinical practice, thus the data were reliable. Secondly, the score was simple and easy to master and has undoubtedly screening values for clinic application.

Finally, the ACES score is a simple, effective, and easy-to-operate screening score. It has a clinical screening effect on unexplained new-onset focal epilepsy, which has a sensitivity of 100%, and has a particular screening value for most patients with AE, and the sensitivity is about 99.2%. In clinical practice, it is necessary to increase the knowledge and testing of thyroid function and rheumatism for patients with suspected symptoms associated to AE. For patients with mild but no obvious neurological symptoms, such as insomnia, muscle stiffness, which may be considered to be side effects of anti-seizure medication, the scope of consultation should be expanded to avoid missing and delaying diagnosis.

## Data Availability Statement

The original contributions presented in the study are included in the article/supplementary material, further inquiries can be directed to the corresponding author/s.

## Ethics Statement

This study was approved by the Ethics Committee of the First Affiliated Hospital of Zhengzhou University. Written informed consent to participate in this study was provided by the participants' legal guardian/next of kin.

## Author Contributions

All authors listed have made a substantial, direct, and intellectual contribution to the work and approved it for publication.

## Funding

This work was supported by the National Natural Science Foundation of China (No. 81771397).

## Conflict of Interest

The authors declare that the research was conducted in the absence of any commercial or financial relationships that could be construed as a potential conflict of interest.

## Publisher's Note

All claims expressed in this article are solely those of the authors and do not necessarily represent those of their affiliated organizations, or those of the publisher, the editors and the reviewers. Any product that may be evaluated in this article, or claim that may be made by its manufacturer, is not guaranteed or endorsed by the publisher.
